# Unnecessary Hysterectomy due to Menorrhagia and Disorders of Hemostasis: An Example of Overuse and Excessive Demand for Medical Services

**DOI:** 10.3389/fphar.2016.00507

**Published:** 2016-12-23

**Authors:** Svetlana M. Djukic, Danijela Lekovic, Nikola Jovic, Mirjana Varjacic

**Affiliations:** ^1^Clinic for Hematology, Faculty of Medical Sciences University of KragujevacKragujevac, Serbia; ^2^Clinic for Hematology, Medical Faculty University of BelgradeBelgrade, Serbia

**Keywords:** hysterectomy, menorrhagia, disorder of hemostasis, medical service use, anemia

## Menorrhagia in clinical medicine

Excessive menstrual bleeding—menorrhagia is a common gynecologic disorder affecting women of reproductive age. Subjectively, menorrhagia is defined as a complaint of heavy cyclical menstrual bleeding occurring over several consecutive cycles (Rönnerdag and Odlind, 1999). Objectively, it can be defined as heavy menstrual bleeding lasting for more than 7 days or resulting in the loss of more than 80 mL per menstrual cycle (ACOG Committee on Practice Bulletins—Gynecology, American College of Obstetricians and Gynecologists, 2001). An objective evaluation of the existence of menorrhagia is not simple. Alkaline hematin technique is completely objective measure (extracting hemoglobin from sanitary wear to assess blood loss), but it is impractical out of controlled research settings. Widely used alternative is the pictorial blood loss assessment chart (PBAC) and this is semiobjective method takes into account the number and the degree of staining of items of sanitary wear used. PBAC is easier to perform than the alkaline hematin technique, yet yields more objective results than self-reporting (Warner et al., 2004). Data from literature suggested that approximately 10% of reproductive-aged women had objective evidence of menorrhagia, but studies based on self-reported information suggested that approximately 30% of women of reproductive age were afflicted with heavy menstrual bleeding (Dilley et al., 2002; Shapley et al., 2004). According to our research, out of 115 women who self-report these excessive menstrual bleeding only 55% had actually verified menorrhagia by PBAC (Djukic et al., 2013). Menorrhagia can happen due to anatomic (uterine fibroids, endometrial polyps, endometrial hyperplasia, and pregnancy), endocrinologic (thyroid and adrenal gland dysfunction, pituitary tumors, anovulatory cycles, polycystic ovarial syndrome, obesity, and vasculature imbalance), iatrogenic (steroid hormones, chemotherapy agents, medications) and organic (organ dysfunction infection, bleeding disorders) abnormality (Vilos et al., 2001; Albers et al., 2004).

## The role of bleeding disorders

Underlying bleeding disorders only have been recognized during the last two decades as a significant etiopathogenetic factor for menorrhagia formation. The available data from the literature show the frequency of hemostasis disorders in women with menorrhagia in the range of 10–20% (El-Hemaidi et al., 2007), whereas some of the most representative studies by Kadir and associates state the information about 17% of patients (Kadir et al., 1998). The reported prevalence of von Willebrands Disease (vWD) as the most frequent among them is ranging from 5 to 20% with an overall estimate of 13%, based on a systematic review (Shankar et al., 2004). The considerable proportion of women with menorrhagia is found to have single coagulation factor deficiencies such as factor XI deficiency (1–4%), carriers of hemophilia A and hemophilia B observed in approximately 1–4% of females with menorrhagia and less common deficiencies of factor I, II, V, VII, X, XI, XIII (Dilley et al., 2001; Mannucci et al., 2004; Philipp et al., 2005; Plug et al., 2006).

**Figure 1:**
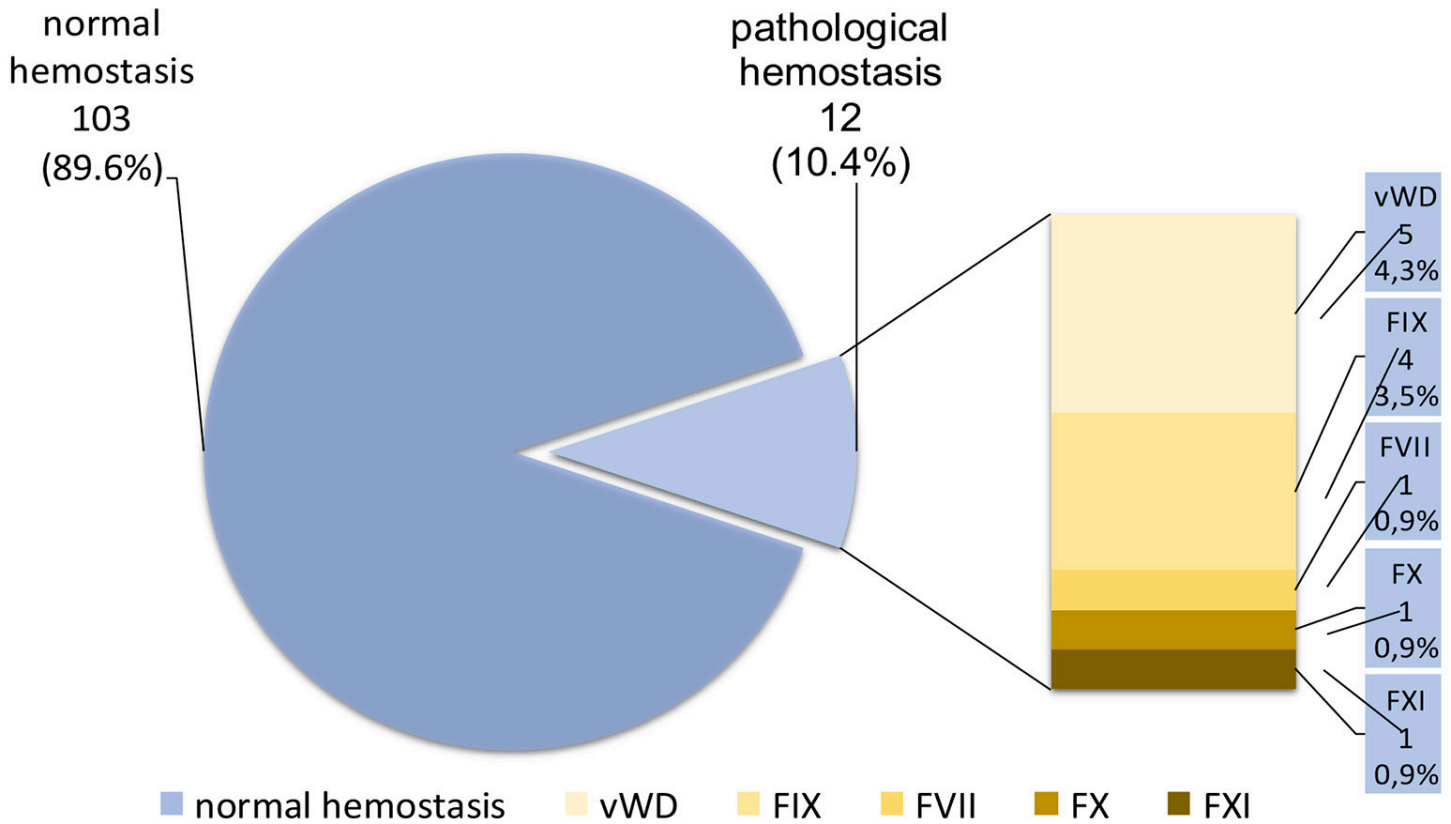
**Frequency of coagulation disorders in Serbian women with symptoms of menorrhagia**. vWD, von Willebrand disease; FVII, factor VII deficiency VII; FIX, factor IX deficiency; FX, factor X deficiency; FXI, factor XI deficiency.

Previous research has shown that in the population of women who suffer from menorrhagia, the frequency of disorders of hemostasis is 17% (Djukic et al., 2009). Analyzing the incidence of certain disorders of hemostasis in previous research is shown that the most commonly disorder is also VWD, although deficiency of factor IX, then deficiencies of factor FVII, X, and XI (Djukic et al., 2013) (Figure 1).

Studies have shown that physicians are not likely to consider a bleeding disorder as a possible cause of menorrhagia. Only 3% of them would refer patients to a specialist and four percent of gynecologists surveyed would consider von Willebrand's disease as a possible diagnosis in women with menorrhagia (Dilley et al., 2002). Anamnestic indicators suggesting an underlying bleeding disorder include menorrhagia since menarche, failed response to conventional management of menorrhagia or family history of a bleeding disorder. In addition, the clinical presentation includes: epistaxis, bleeding of oral cavity or gastrointestinal tract without an obvious anatomic lesion, notable bruising without injury; minor wound bleeding, prolonged or excessive bleeding after dental extraction, unexpected postsurgical bleeding, hemorrhage from ovarian cysts or corpus luteum; hemorrhage requiring blood transfusion; postpartum hemorrhage (especially delayed) (Nicols et al., 2008; Rodeghiero et al., 2009). In a population of women who suffer from heavy menstrual bleeding, disorders of hemostasis are often not recognized. Some studies show that the diagnostic delay from onset of bleeding symptoms can be up to 16 years (Kirtava et al., 2004). As these patients usually do not respond adequately to conventional treatment for menorrhagia, the radical procedures (like a hysterectomy) carry out more often than is necessary. Referral to an attending gynecologist for menorrhagia meant a 43% chance of a hysterectomy (Coulter et al., 1991) and menorrhagia is the major cause for approximately 300,000 hysterectomies per year in the U.S (James et al., 2006). Studies have shown that women with von Willebrand's disease are more likely to undergo a hysterectomy and to have the hysterectomy at a younger age (Kirtava et al., 2003). A randomized comparison of approach with hysterectomy and the levonorgestrel-releasing intrauterine system (IUS) in terms of the quality of life of women with menorrhagia and cost-effectiveness demonstrated that health-related quality of life improved significantly in both the IUS and hysterectomy, but overall costs were about three times higher for the hysterectomy group (Hurskainen et al., 2001). Timely diagnosis and treatment of hemostasis disorders in women with menorrhagia, unnecessary hysterectomy could be avoided. Undiagnosed coagulation abnormalities have effect on women's quality of life. It can cause serious problems such as iron deficiency anemia, complications from surgical procedures, lost work or school time, lifestyle issues, psychological problems (Rae et al., 2013). Anemia is associated with menorrhagia and coagulation abnormalities in women of reproductive age. At least 20 % of women with heavy menstrual bleeding experience anemia (Vercellini et al., 1993). In the local European study of 115 women who reported menorrhagia 53% suffered from anemia (Djukic et al., 2011).

## Excessive hysterectomy and opportunity costs of bleeding disorders medical care

Disorders of hemostasis, especially the ones seldom recognized (Lukes et al., 2005), have a major impact on health-related quality of life, work impairment and health-care costs. So far published data indicate that this field of clinical medicine accounts for a large share of workload for the national health systems, hospital sector and primary care alike (Fraser et al., 2009). Global Burden of Disease Project reports scale of morbidity, mortality (Wang et al., 2016) and disability attributable to this group of illnesses to a great detail (Vos et al., 2016). Associated workload and economic burden of abnormal uterine bleeding was proved to be significant even in highly effective health sectors. The national health expenditure available and current resource allocation strategy varies greatly with mature Western (Jakovljevic, 2016) and top performing health markets representing different historical legacies (Jakovljevic et al., 2016a). However, regardless of these large geographical diversity, willingness to pay threshold in bleeding disorders remains an issue for public debate in many countries (Eastaugh, 2000). In case of hemophilia and Von Willebrand's illness as the most frequent conditions, thorough studies on cost-effective procedures are available (Miners et al., 1998). Due to bold pharmaceutical innovation in this area a variety of drugs have been evaluated as well (Goudemand, 1999). However, particularly concerning interventions are presented by the surgical procedures that might have been avoided. Once performed, hysterectomia in women of child bearing age, in the Era of low fertility, (Jakovljevic and Laaser, 2015) could mean a life time decision affecting family planning and core life goals (Cloutier-Steele and West, 2003; Sardeshpande, 2014).

Certain lack of awareness of the potential of hemostasis disorders to cause abnormal bleeding is clearly present in the clinical gynecology. Besides there is a substantial need to develop more reliable clinical tools for the objective assessment of excessive menstrual bleeding. These circumstances lead to the underdiagnosed cases and suboptimal treatment of women with bleeding disorders, including unnecessary hysterectomy (Ranson and John, 2002). In the US setting it has already been proven that hysterectomy imposes a significant burden on the national hospital sector (Easterday et al., 1983). Furthermore, it is known that other major gynecological interventions such as cesarean section are greatly overused and the major obstacle to delivering universal health coverage nationwide (Gibbons et al., 2010). It was even since the 1980s that elective hysterectomy was disputed as an exemplary non-necessary surgical intervention in many clinical cases (Travis, 1985). Economic consequences of abnormal uterine bleeding attracted attention in academic research even in more recent years (Liu et al., 2007). Overlooked evidence in clinical interventions makes serious ramifications in terms of excessive consumption of non-necessary medical services and incurred additional costs of care (Palmer et al., 1986). In most situations attending physician is unaware of the existing evidence to guide his/her decision toward far less risky pharmacological treatment of bleeding disorder (Chang et al., 2003). Thus, such excessive surgery falls into the so called “supplier induced demand” phenomenon (Labelle et al., 1994). One of the possible approaches to tackle this inefficiency might be to invest into the capacity building and raise clinicians' consciousness about more cost-effective and less risky procedures (Jakovljevic et al., 2016b). Among other solutions has been proposed an alternative treatment strategy of deploying levonorgestrel-releasing intrauterine system vs. hysterectomy for treatment of menorrhagia (Hurskainen et al., 2001). In risky pregnancies and many other associated conditions there is documented evidence of significant inequality in incremental cost-effectiveness ratios even among the standard gynecological treatments (Jakovljevic et al., 2008). Such inequalities in ICERs measured in a sound methodological framework indicate that clinical physician's should be more acquainted to deal with health economic evidence. History of health economics taught us that even minor niche for improvement in clinical decision making could mean a lot for the social opportunity cost of potentially misleading reasoning (Jakovljevic and Ogura, 2016). This peculiar insight from the gynecology surgical practice gives an excellent hints toward serious and hidden causes of excessive demand for medical services. Opportunity costs of lost opportunity for conventional treatment via pharmacological protocols, here might mean incurring higher costs of medical care in the short run (Jakovljevic et al., 2016c). However, potential for long term savings is obvious. Particularly keeping in mind that population aging is the cornerstone demographic landmark of our time (Jakovljevic and Ogura, 2016). Preserving the fertility choice for young women who might be willing and capable to sustain at least one or one more child in their lifetime is precious. It's not only a blessing for the individual family. It is a blessing for most modern day societies whose demographic dividends have long forgone (Pandey, 2015). Medical decision crossroads where such a deep choices depend on estimate and knowledge of single attending physician should be given far more attention in a foreseeable future.

## Author contributions

SD made conception of the work, data collection and interpretation. DL made data collection and interpretation. NJ made data collection. MV made conception of the paper, critical revision of the article and final approval.

### Conflict of interest statement

The authors declare that the research was conducted in the absence of any commercial or financial relationships that could be construed as a potential conflict of interest.
